# Are nutrients retained by river damming?

**DOI:** 10.1093/nsr/nwaa073

**Published:** 2020-04-18

**Authors:** Jack J Middelburg

**Affiliations:** Department of Earth Sciences, Utrecht University, The Netherlands

Rivers are dammed for hydropower, flood control, navigation or water storage for human use. Globally, about half of the rivers are impacted by damming [1], with major consequences for biodiversity, fish migration, primary production, greenhouse gas emissions and retention of nutrients [2,3].

The traditional view is that dam closure leads to an increase in water residence time, settling of suspended particles and improved light conditions. This amplifies primary productivity and thus the stripping of dissolved nutrients from the water while transiting [2]. The imported and locally produced organic matter settles to the sediment, where part of it is mineralized to carbon dioxide, methane, phosphate and ammonium, and another part is temporarily or permanently buried. This nutrient transfer from transiting river water to the sediment causes removal of nutrients via burial and dinitrogen gas production by denitrification and Anammox. This retention from dam construction reduces nutrient supply to downstream ecosystems and thus partly alleviates eutrophication.

This retention of nutrients scales with water residence time, and most mathematical models for regional and global nutrient transport along the aquatic continuum from streams to estuaries are based on this concept [2,3].

However, this simple depiction has its limitations. Nutrients enter reservoirs in various forms (e.g. organic and inorganic, particulate and dissolved, unreactive and reactive) and intense biogeochemical cycling within reservoirs may modify the forms of and ratios between the nutrients with consequences for downstream biodiversity and ecosystem functioning (e.g. replacement of diatoms by green algae or cyanobacteria). In this issue, Chen *et al*. [4] convincingly show that a series of reservoirs in the upper Mekong river increased rather than decreased the bioavailability of nitrogen and phosphorus downstream. Specifically, non-bioavailable particulate phosphorus was retained while soluble reactive P and bioavailable particulate P increased. Moreover, dissolved inorganic nitrogen entered the reservoir in the form of nitrate, but exited as ammonium. These changes in N and P speciation impacted the functioning of downstream ecosystems, for example, via changes in the dominant phytoplankton groups (diatoms vs. green algae) and food webs (e.g. fishery yield) [4], and likely impacted microbial processes (nitrification) as well. This enhancement rather than attenuation of nutrient supply to downstream rivers can be attributed to water-column stratification, development of low-oxygen conditions in bottom waters and the reservoir exiting at depth where low-oxygen, phosphate and ammonium-rich water has accumulated. The Chen *et al*. [4] study raises several questions: what is the footprint of individual and a series of reservoirs? Can we predict the occurrence of hypoxia (and thus P and N release) based on reservoir morphology (e.g. size and water depth)? The biogeochemical functioning of reservoirs is an emerging property that varies with their age. Initially, reservoirs may emit greenhouse gases and retain nutrients, but long-term accumulation of nutrients may induce legacy effects. Old reservoirs with large stocks of nutrient-rich sediment may release nutrients and in this way, counteract environmental policy measures. Evidently, the diversity in reservoirs complicates the development and use of predictive models based on water residence time only and implies the need for long-term field observations and detailed process studies.
